# State-Based Markers of Disordered Eating Symptom Severity

**DOI:** 10.3390/jcm9061948

**Published:** 2020-06-22

**Authors:** Matthew Fuller-Tyszkiewicz, Isabel Krug, Joshua M. Smyth, Fernando Fernandez-Aranda, Janet Treasure, Jake Linardon, Rajesh Vasa, Adrian Shatte

**Affiliations:** 1 School of Psychology, Deakin University, Geelong, VIC 3220, Australia; jake.linardon@deakin.edu.au; 2 School of Psychological Sciences, University of Melbourne, Melbourne, VIC 3010, Australia; Isabel.krug@unimelb.edu.au; 3 Department of Biobehavioral Health, The Pennsylvania State University, University Park, PA 16802, USA; jms1187@psu.edu; 4 Department of Psychiatry, University Hospital of Bellvitge-IDIBELL and CIBERobn, 08907 L Hospitalet De Llobregat, Spain; ffernandez@bellvitgehospital.cat; 5 Department of Psychological Medicine, Institute of Psychiatry, Psychology & Neuroscience, King’s College London, London SE59RJ, UK; janet.treasure@kcl.ac.uk; 6 Applied Artificial Intelligence Institute, Deakin University, Geelong, VIC 3220, Australia; rajesh.vasa@deakin.edu.au; 7 School of Science, Engineering, Information Technology, & Physical Sciences, Federation University, Berwick, VIC 3806, Australia; a.shatte@federation.edu.au

**Keywords:** state body image, experience sampling, disordered eating, within-person dynamics, ecological momentary assessment

## Abstract

Recent work using naturalistic, repeated, ambulatory assessment approaches have uncovered a range of within-person mood- and body image-related dynamics (such as fluctuation of mood and body dissatisfaction) that can prospectively predict eating disorder behaviors (e.g., a binge episode following an increase in negative mood). The prognostic significance of these state-based dynamics for predicting trait-level eating disorder severity, however, remains largely unexplored. The present study uses within-person relationships among state levels of negative mood, body image, and dieting as predictors of baseline, trait-level eating pathology, captured prior to a period of state-based data capture. Two-hundred and sixty women from the general population completed baseline measures of trait eating pathology and demographics, followed by a 7 to 10-day ecological momentary assessment phase comprising items measuring state body dissatisfaction, negative mood, upward appearance comparisons, and dietary restraint administered 6 times daily. Regression-based analyses showed that, in combination, state-based dynamics accounted for 34–43% variance explained in trait eating pathology, contingent on eating disorder symptom severity. Present findings highlight the viability of within-person, state-based dynamics as predictors of baseline trait-level disordered eating severity. Longitudinal testing is needed to determine whether these dynamics account for changes in disordered eating over time.

## 1. Introduction

Eating disorders (EDs) are common, with a combined estimated prevalence of up to 16% across the key subtypes of anorexia nervosa, bulimia nervosa, and binge eating disorder [1,2]. Although important differences exist between these subtypes of eating disorders, food-related concerns are common to all and their differences exist primarily along dimensions of weight status, amount of food consumed, appearance-related concerns, and compensatory behaviors in response to weight goals or food consumption [3]. These ED characteristics can be conceived in both state and trait terms. The trait perspective focuses on severity and persistence of ED symptoms (e.g., number of instances of binge eating per week for 3–6 months). In contrast, at a state level, one may evaluate ED symptoms as discrete events, such as binge episodes, purging, or temporary spikes in body dissatisfaction.

The dominant paradigm for risk detection is prospective studies of stable, trait-like predictors of EDs [4], with ED operationalized in terms of persistence of symptoms (i.e., a trait perspective) rather than as fleeting instances of ED cognitions and behaviors in daily life. To date, within-person dynamics evaluated at the state level have been used to predict onset of ED episodes. With few exceptions [5,6,7], state-level predictors and their within-person dynamics have not been used to predict current or future ED status at the trait level. The absence of literature on state-based predictors of trait-level ED status is surprising given that a range of identified trait-level risk factors, such as emotion dysregulation, rumination, and negative affectivity [4,8,9], also have state-based properties. Drawing upon cognitive and emotion-focused models of ED onset and maintenance, the present study tests several plausible state-based candidates for statistical prediction of current ED status.

### 1.1. Cognitive and Emotional Influences on ED Symptom Progression

Cognitive models emphasize the facilitatory roles that biased information processing and well developed appearance- and food-related schemas have in maintaining and exacerbating ED symptoms [10]. It is argued that individuals who have—or are at elevated risk of developing—an ED prioritize attention towards food- and appearance-related information. Of concern, this focus seems to be more strongly devoted to negative aspects of their appearance that fuel body dissatisfaction and dieting efforts at the expense of attention or willingness to process information that may disconfirm their distorted appearance-related thoughts and feelings [11].

Accumulated evidence from state-based studies supports this notion of cognitive bias, demonstrating that individuals with elevated trait eating pathology (including heightened trait body dissatisfaction) more frequently engage in state-based upwards appearance comparisons (i.e., comparisons to an individual one considers more attractive) [12] Such comparisons have been consistently linked to subsequent increases in state body dissatisfaction [13]. Individuals with elevated trait body dissatisfaction or eating pathology are also more likely to engage in exercise for appearance-related reasons [5]. Unlike exercise engaged in for fitness reasons, appearance-motivated exercise tends to increase—rather than decrease—state body dissatisfaction post-exercise [14,15].

Recent reviews have also highlighted dysfunctional emotion processing as a potential risk factor for EDs [8,9]. At the trait-level, individuals with an eating disorder tend to report higher levels of ruminative tendencies (i.e., greater fixation on negative thoughts) [9], which echoes findings from attentional bias tasks [11]. Individuals with an ED also exhibit more elevated trait-levels—relative to non-ED individuals—on measures of emotion dysregulation. Individuals with an ED report more frequent engagement with maladaptive coping strategies such as disengagement, avoidance, and distraction, exhibit lower confidence in their ability to handle negative emotions when they arise and report greater desire to avoid beneficial contexts if there is a risk of negative emotions [8]. Findings at the state-level broadly support the role of negative emotions in ED symptom expression. Heightened negative mood states have been shown to precede binge episodes [16], consistent with the notion that binge eating serves as a distraction to reduce negative emotions (e.g., [17]). Although there is some evidence to suggest that binge eating reduces negative mood and thus reinforces the behavior, other findings suggest that negative mood may intensify following a binge and is only reduced after subsequent compensatory behaviors, such as purging the food one has consumed [16]. It is also clear that individuals with elevated trait eating pathology more regularly experience negative mood states (both in general and in relation to their appearance), and that they are slower to down-regulate these negative states once initiated [9,11]. For individuals with poor coping ability, this extended period of heightened negative mood may lengthen the risk window for deployment of distraction-based, maladaptive coping strategies, such as binge eating or substance use [17]. 

Interestingly, several lines of evidence highlight potential feedback loops between negative mood and ED cognition and behaviors. In addition to negative mood as antecedent and consequence of binge episodes (e.g., [18]), it has been shown that some individuals are more likely to engage in upward comparisons when they are dissatisfied with their appearance [19], and that such comparisons increase this dissatisfaction [12]. Similarly, dietary restraint can lead to overeating [20,21], which in turn promotes further dieting to offset caloric intake [22]. Emotional disturbances seem central to these feedback loops, and may explain how mood-related risk factors intensify over time in a way that might perpetuate and exacerbate ED symptom severity and frequency. This cycle, which may be experienced within-person yet differ across individuals in presence and magnitude, may thus help to identify who is at risk of transitioning from subclinical to clinical ED levels.

### 1.2. State-Level Data Predicting Trait-Level ED Severity

To date, only a handful of studies have used state-based variables to predict trait-level ED symptom severity [6,7,14], and all of these have used a measure of ED captured in the same span of time as the state-based data rather than prospectively. All three of these studies found that state average body dissatisfaction (i.e., one’s mean level across repeated assessments of state body dissatisfaction) was a significant correlate of ED severity. Fuller-Tyszkiewicz et al. and Melnyk et al. showed that this state average predicted ED severity, after controlling for trait body dissatisfaction. Melnyk et al. found variability around state body dissatisfaction mean was also predictive of ED severity, but this finding did not replicate in Fuller-Tyszkiewicz et al.’s (2018) study.

Despite some encouraging findings of association between state risk factors and trait ED, the range of state-based risk factors used in these prior studies was narrowly focused on state body dissatisfaction. Given evidence at the trait level of the role of negative affect in EDs, the predictive value of negative mood warrants testing. More sophisticated within-person dynamics involving negative mood (both general and in relation to body image) may lead to further understanding of state-based markers of EDs. First, in keeping with emotion regulation-based models of ED, further research should explore persistence of negative mood in daily life once initiated. Recent work by Fuller-Tyszkiewicz et al. (2020) used a cutoff of 5 and above (out of 10) to indicate elevated state body dissatisfaction, and then derived a measure of persistence by evaluating change in state body dissatisfaction levels by the subsequent timepoint (~1–2 h later) [23]. The authors found that for individuals with elevated appearance-related attentional bias, heightened state body dissatisfaction tended to decrease by a smaller magnitude than for individuals with a lower level of this attentional bias. Second, insofar as negative mood states precipitate and arise from ED-related cognitions and behaviors in daily life, the magnitude of these state-based relationships may also differentiate individuals with an ED from those without an ED.

### 1.3. The Present Study

The present study further explores state-based predictors of trait-level ED symptom severity, incorporating a wider range of state-based variables than considered in previous work [6,7,14]. Specifically, we explore the individual and combined predictive value of three types of person-specific, state-based characteristics (state averages for ED-related variables, persistence of mood disturbances, and magnitude of within-person associations among these ED-related variables) for trait ED symptom severity.

Given the exploratory nature of this full model, we do not make hypotheses about which of these state-based characteristics will have the strongest association with trait ED symptom levels. Furthermore, as our intention was to evaluate mood-based markers that may exist along the continuum of trait ED symptom severity, the decision was made to limit state-based measures to constructs that may be common even among individuals at subclinical levels of trait ED symptom severity: negative mood, body dissatisfaction, upward appearance comparisons, and dieting efforts. Although binge eating and purging efforts are likely related to mood-based disturbances [16], these behaviors occur less frequently in daily life [20,24,25] and may be absent for many individuals with low-level trait ED symptoms. The strength of associations between negative mood and these less common ED behaviors may better distinguish later stages of the trait ED symptom severity continuum.

## 2. Methods

### 2.1. Participants

The present sample drew on data from two pre-existing studies [5,14]. In both studies, participants were recruited via on-campus recruitment efforts (labs, lectures, gyms, eating areas, and discussion boards) at two Australia-based universities (University of Melbourne and Deakin University), as well as via social media and online forums (e.g., Facebook and Reddit). Participation was limited to women in recognition that the bulk of prior ecological momentary assessment (EMA; alternatively referred to as ambulatory assessment or experience sampling method) studies have sampled women [12]. Although the parent studies did not exclude participants with low numbers of EMA surveys completed, we applied a 50% lower limit in the present analyses to ensure higher amounts of data across all participants in the sample to aid analysis. This 50% lower limit was consistent with recent studies in the field (e.g., [13,26]).

These inclusion criteria resulted in a final sample of 260 women. The sample had a mean age of 22.03 years (SD = 5.80) and a mean body mass index (BMI) categorized as normal (M = 22.51, SD = 4.57). The demographics of this sample are reported in Table 1.

### 2.2. Materials

#### 2.2.1. Baseline Measures

##### BMI

Participants provided self-reported weight and height to permit calculation of BMI.

##### Trait Disordered Eating Symptom Severity

The 26-item version of the eating attitudes test (EAT-26; [27]) was used to evaluate trait disordered eating symptom severity. Items were rated on a 6-point scale (0 = never to 5 = always), and scores were added to create a total index of severity. Higher scores reflected greater levels of trait eating pathology. In the present study, this total score was used as a continuum in recognition of dimensional models of eating disorders (e.g., [5]) and also using several cut-points supported by prior literature. The standard cutpoint of 20 was used to differentiate individuals without an ED from those who may be at risk [28]. However, as this cutpoint may lump subthreshold ED cases with non-ED cases, we also applied a lower cutpoint of 11, which has been shown to reduce false negative rates [29].

#### 2.2.2. State-Based Measures

##### Body Dissatisfaction

State body dissatisfaction was measured with the item “How satisfied are you with your appearance right now?” on a 11-point scale, ranging from 0 (completely dissatisfied) to 10 (completely satisfied). The item was reverse-coded with higher scores suggesting greater state body dissatisfaction. This single item approach has been used previously by studies investigating state body satisfaction (e.g., [12,30]).

##### Negative Mood

Negative mood was evaluated by asking participants to rate how happy they were right now, with response options range from 0 (very unhappy) to 10 (very happy). Consistent with past uses of this measure (e.g., [20]), scores were reverse coded so that higher scores reflected a negative emotional state.

##### Dietary Restraint

Dieting behavior was measured using two items, “Did you consciously restrict food intake since the last survey?” and “Did you skip a meal since the last survey?”, with response options 0 = no, 1 = yes. Item scores were combined and rescaled so that 0 = no for both items, 1 = yes for at least one of the two items. This binarized approach is consistent with previous studies assessing disordered eating behaviors using EMA (e.g., [20,21,31]).

##### Appearance Comparison

Participants were asked to indicate the level of body comparison behavior they engaged in since the last time they were signaled on an 11-point scale, ranging from 0 (no body comparisons) to 10 (constantly making body comparisons). If participants responded with a value greater than zero, they were then prompted to indicate how they compared to their most recent comparison target: (1) much worse, (2) worse, (3) the same, (4) better, or (5) much better. A separate category was made (no comparison) when the prior question was answered with a value of 0. Given our interest in the relationship between upward comparisons and state body dissatisfaction, the direction of appearance comparison was recoded as upward comparisons (worse, much worse) = 1 and all other responses = 0. This dichotomous approach has been used previously (e.g., [24]).

### 2.3. Procedure

Following ethics approval from Deakin University’s Human Research Ethics Committee (DUHREC 2015-008, DUHREC 2016-128) and the University of Melbourne’s ethics committee (HREC 1544167.1, HREC 1646111.1), the study was advertised via social networking sites (Facebook, Gumtree, etc.) and through advertising in lectures, labs, and common meeting places on campus at two Australian universities. This advertising included a weblink that directed potential participants to a plain language statement about the study. Those who consented (via button click) to participate then completed the baseline measures and followed instructions to download a smartphone app (Instant Survey; Richardson, 2015a, 2015b) [32,33] for the EMA phase. The app generates a random ID for participants, which they are asked to enter into the baseline survey to enable data linkage across the two phases of participation. On the day following download, the smartphone app commenced, signaling participants to complete brief surveys of state-based measures 6 times daily for 7-10 days. Surveys were set to signal at semi-random intervals spaced in 1–2 h blocks to ensure sampling across the whole day from 9 am to 10 pm.

### 2.4. Data Analytical Plan

#### 2.4.1. Extraction of State-Based Dynamics

Multilevel models were run in Mplus version 8 to derive and extract person-specific estimates of state-based dynamics. State averages for negative mood, body dissatisfaction, dietary restraint, and upward appearance comparisons were calculated as person-specific means for these variables. Persistence of negative mood states was operationalized as difference scores calculated by subtracting current negative mood (or state body dissatisfaction) rating from the previous mood (or state body dissatisfaction) rating within the same day; these difference scores were treated as an outcome variable in subsequent multilevel models which sub-setted the data, only using estimates of difference in mood/body dissatisfaction scores when the initial score was higher than 5/10. This threshold of >5 to identify “high” negative mood states was based on prior analyses with this dataset, which showed that this cutoff provided strong differentiation between individuals with a probable eating disorder and those without [14]. Time lag between observations was included as a covariate, and the intercepts from these models were saved as indices of tendency for individuals to remain in negative mood states following initial spikes.

Individual differences in magnitude of person-specific associations were also derived from random effects in multilevel models for relationships in which (1) state body dissatisfaction at time *t* predicts subsequent upward appearance comparisons and dietary restraint at time *t* + 1 (controlling for comparisons and dietary restraint scores reported at time *t*), (2) negative mood at time *t* predicts subsequent dietary restraint at time *t* + 1 (controlling for dietary restraint at time *t*), (3) upward comparison behavior at time *t* predicts state body dissatisfaction at time *t* (controlling for state body dissatisfaction scores at time *t* − 1), and (4) dietary restraint at time *t* predicts negative mood at time t (controlling for negative mood at time *t* − 1). Please note that given the wording of the mood variables (right now) and the behavioral variables (since last signal), behavioral variables at time *t* predicting mood at time *t* should constitute a prospective association.

#### 2.4.2. State and Trait Associations

Bivariate correlations were conducted initially to evaluate the extent to which baseline (trait body dissatisfaction, age, and BMI) and the state-level dynamics listed above correlate with trait disordered eating symptom severity. Subsequently, regression models were conducted to show the combined contribution of the state-based dynamics for predicting disordered eating. These regressions were run three times: once with disordered eating as a continuous variable, using multiple regression, and once each with trait disordered eating as a categorical variable (using 11 and 20 as the cutpoints, respectively) within a logistic regression framework. All significance testing was set at *p* < 0.05 (two-tailed). Missing data for the bivariate correlations and regression models were handled by using full-information maximum likelihood estimation instead of imputing. For the EMA data extraction, only participants who completed at least 50% of the EMA timepoints, and only timepoints that these participants completed, were used in the analysis.

## 3. Results

### 3.1. Descriptive Statistics and Correlations

Table 2 provides descriptive statistics for variables intended for regression analyses. For the sample overall, the mean trait eating pathology level was 10 (SD = 10.39; range 0–52). In total, 77 individuals (29.6%) had EAT26 scores above 11, whereas 34 individuals (13%) had EAT26 scores above 20. Average state body dissatisfaction scores (on a 0–10 scale) were moderate, whereas average state negative mood scores were marginally lower. Upward appearance comparisons were reported for 26% of EMA reports, while dietary restraint behaviors were less common (12% of all reports). When state negative mood and body dissatisfaction levels were high (>5) at a given time point, the tendency was for these states to improve by around 1.5 units by the next time point (~1–2 h later). There was, however, considerable between-person variability around these average effects (as evidenced by SD values in Table 2), suggesting that some individuals tended to remain at high levels of these state-based variables. Associations among state-based constructs showed that, for the sample overall, state body dissatisfaction tended to increase the likelihood of upward comparisons and dietary restraint, negative mood states increased the likelihood of dietary restraint, and that engaging in dietary restraint was associated with reduced state body dissatisfaction and negative mood. Engagement in upward comparisons was associated with an increase in body dissatisfaction.

Bivariate correlations showed that trait eating pathology was significantly and positively associated with average state body dissatisfaction (*r* = 0.48, *p* < 0.001) and negative mood (*r* = 0.37, *p* < 0.001), frequency of engagement in dietary restraint (*r* = 0.45, *p* < 0.001) and upward appearance comparisons (*r* = 0.61, *p* < 0.001), persistence of state negative mood (*r* = 0.20, *p* = 0.004), persistence of state body dissatisfaction (*r* = 0.30, *p* < 0.001), and strength of within-person association between state body dissatisfaction and subsequent appearance comparison behavior (*r* = 0.58, *p* < 0.001). Trait eating pathology was also significantly negatively related with magnitude of person-specific effects of state negative mood and body dissatisfaction on subsequent dietary restraint behavior (*r* = −0.38, *p* < 0.001 and *r* = −0.42, *p* < 0.001, respectively). Trait eating pathology was unrelated to magnitude of person-specific effects of dietary restraint on subsequent state negative mood and body dissatisfaction (*r* = −0.10, *p* = 0.112 and *r* = −0.07, *p* = 0.246, respectively), and the magnitude of person-specific effect of appearance comparison behavior on subsequent state body dissatisfaction (*r* = −0.09, *p* = 0.398).

### 3.2. Regression Analyses

Table 3 provides a summary of coefficients for statistical prediction of trait eating pathology, as a continuous variable (left-hand side) and categorical variable with cut-offs of >11 and >20 for a “possible ED” (middle and right-hand side, respectively). In combination, the state-based constructs accounted for a substantial amount of variance in trait disordered eating symptom severity (*R*^2^ = 0.34 for trait ED symptom severity as a continuous construct and *R*^2^ = 0.39–0.43 for trait eating pathology as a categorical outcome variable).

For trait eating pathology as a continuous outcome, only the average for state body dissatisfaction made a significant unique contribution to prediction. For trait eating pathology as a categorical outcome, average state levels for both body dissatisfaction and negative mood were significant unique predictors whether a cutoff of 11 or 20 was used, whereas the magnitude of person-specific effects of state body dissatisfaction and negative mood on subsequent dietary restraint were only significant predictors for the categorical model with a cutoff of 20. The finding for state negative mood in this full model reverses the sign (negative association) found via bivariate correlation (positive association).

## 4. Discussion

A wide range of biological, psychological, and environmental risk factors for EDs have been theorized and empirically tested [4,34,35]. Although many of these putative risk factors could be operationalized in state or trait terms, limited research attention has been given to assess how state-based dynamics may contribute to prediction of ED symptom severity and progression. Present findings add to limited earlier work [5,6,7] by demonstrating that state-based, mood-related dynamics that unfold quickly in daily life help to distinguish individuals with elevated trait ED symptom severity. The pattern of bivariate correlations suggests that state averages for negative mood, body dissatisfaction, dieting, and appearance comparisons, smaller reductions from temporary spikes in negative mood states (both general and in relation to body dissatisfaction), and magnitude of person-specific effects of body dissatisfaction and negative mood on appearance comparisons and dieting behaviors may be daily life indicators of trait eating pathology. Further, in combination, these fast-moving, state-based variables contributed substantially to statistical prediction of trait eating pathology and may thus have some predictive value whether in combination with, or separate from, trait-level predictors of ED symptom severity.

The finding that individuals with elevated trait eating pathology were more likely to engage in upward comparisons when experiencing state body dissatisfaction is consistent with the notion that trait-level ED symptoms of these individuals may arise from selective attention towards someone they perceive as more attractive than themselves, which serves to make them feel worse about themselves (e.g., [10,11]). Prior studies have consistently shown that upward appearance comparisons predict increases in state body dissatisfaction regardless of level of trait eating pathology [12]. As such, the lack of predictive effect of the magnitude of the appearance comparison to body dissatisfaction relationship on trait eating pathology should not be interpreted as these individuals failing to have a body dissatisfaction increase post-comparison. After all, for the sample overall, a positive association was found for upward comparisons and subsequent state body dissatisfaction increases. Rather, we argue that this pattern of findings may instead signal that what differentiates individuals with trait eating pathology is that they exhibit a bidirectional relationship between state body dissatisfaction and appearance comparison in daily life. Such a feedback loop may help to explain why individuals with elevated trait eating pathology engage in these comparisons more regularly, and it could also explain intensification of trait body dissatisfaction (and, in turn, trait eating pathology) over time.

Although elevated body dissatisfaction and negative mood states were more likely to lead to instances of dietary restraint for the sample overall, the magnitude of these within-person effects were actually negatively associated with trait-level ED symptom severity. These surprising findings may be explained in several ways. Plausibly, as individuals with an eating disorder may routinely engage in dieting as an intentional and long-standing strategy to improve their appearance, instances of dieting may be less subject to spikes in negative mood. Alternatively, over time, individuals with an eating disorder may develop greater sensitivity to aversive thoughts and emotions, and may thus have a lower threshold for mood states to influence dieting behaviors. This latter conjecture is consistent with findings that distress intolerance is common for individuals with an eating disorder [36], and that even low levels of negative mood can predict onset of ED behaviors in daily life [21]. The veracity of these explanations requires longitudinal follow-up to determine whether the strength of these person-specific associations remain stable or change over time as individuals’ ED symptom severity changes. There is some support for this suggestion of changing magnitude of person-specific effects as symptoms intensify within the context of depression [37].

### Limitations and Future Research Directions

Present findings should be placed within the context of key study limitations. First, although state-based predictors accounted for substantial variance in trait ED symptom levels measured at baseline (i.e., prior to the state assessment period), the present study design does not permit evaluations of whether these state-based measures can predict change in trait ED symptoms over time. Longitudinal confirmation of these observed effects is necessary to further establish the prognostic value of state-based, person-specific data patterns for predicting trait ED symptom trajectory. Second, sampling was limited to women, and the generalizability of current findings to males remains unclear. This remains an area in further need of investigation as relatively few EMA-based body image and disordered eating studies have included men [12]. Further, adjustments were not made in analyses for demographic influences such as age or BMI. Third, eating pathology was based on a self-report measure (the EAT-26), and not confirmed via more objective means such as clinical interview. Furthermore, results were slightly stronger when using a higher threshold for classifying probable ED cases. As such, the predictive utility of these state-based predictors may change across the trait ED symptom severity spectrum [38,39]. Future testing could more comprehensively evaluate whether these predictors differentiate asymptomatic from subclinical, subclinical from clinical, and also across different types of EDs. As an early attempt to explore the predictive value of state-based dynamics on trait ED symptom severity, we limited focus to state-based constructs that were most defensible in terms of the current state-based literature and recognition that the sampling approach would capture individuals across the trait spectrum of ED symptom severity. Further testing of other appearance-related constructs, such as engagement in fat talk, excessive exercise, and appearance self-monitoring, and ED behaviors that are less common in the general population, such as binge-eating and purging, should be considered to more comprehensively model the influence of state-based dynamics on trait-level ED symptom progression.

Hamaker (2012) warns that predictive effects of inter-individual differences in a target variable may not correspond to intra-individual changes over time [40]. Although the present findings are encouraging in showing clear and moderate-to-large correlations between person-specific, it is unclear which of these predictors may be best placed to predict change in ED symptom severity over time. As argued by several researchers (e.g., [38,39]), prediction of onset of trait ED symptoms is different from prediction of trait ED symptom maintenance or intensification, and we should not be surprised to find that different predictor sets work best for prediction of different stages of ED symptom progression.

Although state-based averages tended to have the strongest correlation with current trait-level ED symptom severity in the present study, this may be little more than concordance among symptoms expected to pair together to varying degrees based on one’s current ED status. Dynamics such as an individual’s capacity to bounce back from a momentary spike in negative mood states vs persistence of negative mood, or strength of person-specific associations between mood disturbances and ED-related thoughts and behaviors may turn out to be more important predictors longitudinally of changes in ED status. Indeed, if individuals begin to experience more frequent episodes of negative mood states that persist for longer periods of time, this may serve to undermine their self-efficacy for coping with stressors and may be felt as an intense emotional experience that encourages distracting, though self-defeating, behaviors such as binge eating [17]. Furthermore, findings supportive of a potentially bidirectional association between state body dissatisfaction and appearance comparisons may constitute a feedback loop that serves to intensify both over time. Escalation of these predictors of ED behaviors may, in turn, potentiate more regular engagement in ED behaviors.

We have several key recommendations for future research into prediction of ED symptom progression. First, the strength of present correlations and growing evidence of predictive value of person-specific dynamics for predicting transition in other mental illnesses (e.g., [41]) is encouraging for the ED field, and should be followed up with longitudinal investigations that evaluate (1) the potential stability of person-specific dynamics over time, and (2) the capacity of these person-specific dynamics to predict change in ED symptom severity. Second, we recommend that such longitudinal investigations should blend fast-moving, state-based risk factors and slow-moving, trait-based risk factors into a comprehensive model. This should not only include variables that are clearly best represented at a given time scale (e.g., personality as a slow-moving risk factor and emotion dynamics as fast-moving) but should also consider the same construct measured at different time scales [42,43]. In so doing, the incremental predictive value of testing a construct at its optimal time scale can be evaluated, enabling researchers to judge possible trade-offs between accuracy and complexity of data capture. Third, although the focus in the present study was on the predictive value of state-based data captured with EMA, a comprehensive predictive model might also consider other non-traditional risk factors for longitudinal investigation. For instance, this broader modelling might include experimentally induced effects such as attentional bias indices from lab tasks and passive data capture such as logs of social media use, physical activity, and geo-location to inform eating and socialization habits [44].

Ultimately, if further studies are able to demonstrate the incremental predictive validity of state-based measures for disordered eating, there are clear implications for how we assess for ED risk and screen for current symptom level. Whereas the bulk of existing risk factors are measured in a trait-like manner, EMA-based assessments may provide a viable addition to screening and prediction of ED progression, including prediction of relapse following treatment. Research has started to unlock this potential for other psychological conditions [45,46,47] but could also extend to the realm of eating disorders.

To conclude, in the present study, we advocate for consideration of state-based, person-specific dynamics as historically overlooked yet potentially relevant risk factors to enhance prediction of trait ED symptom severity. While present findings clearly show correlations between these person-specific dynamics and current ED symptom status, we identify specific areas for further testing with longitudinal designs that are needed to confirm the predictive utility of these EMA-derived measures.

## Figures and Tables

**Table 1:** Demographic statistics for sample (*n* = 260).

Variable	M	SD	*n*	%
Age	22.03	5.80		
BMI	22.51	4.57		
Education				
High school			164	63.6%
Diploma			25	9.7%
Bachelor’s degree			53	20.5%
Postgraduate degree			16	6.2%
Ethnicity				
Asian			167	64.7%
Caucasian			69	26.7%
Other			22	8.5%
Paid employment (yes)			135	52.3%

*Note*. Several cases had missing data for categorical variables. Percentages are calculated based on complete cases only and do not add to 260 participants.

**Table 2:** Descriptive statistics for model variables (*n* = 260).

Variable	M	SD	%
ED severity^	10.00	10.39	
Average state body dissatisfaction	4.28	1.84	
Average state negative mood	3.90	1.56	
Frequency of state dietary restraint reports			12.1
Frequency of state upward comparisons			26.3
Persistence of state body dissatisfaction	−1.42	0.87	
Persistence of state negative mood	−1.65	0.69	
State body dissatisfaction → upward comparisons	0.07	0.02	
State body dissatisfaction → dietary restraint	0.06	0.03	
State negative mood → dietary restraint	0.19	0.11	
State dietary restraint → body dissatisfaction	−0.15	0.21	
State dietary restraint → negative mood	−0.08	0.16	
State upward comparison → body dissatisfaction	0.36	0.30	

*Note*. ^ ED severity is based on EAT26 total scores. ED = eating disorders.

**Table 3:** Results of regression analysis predicting disordered eating symptom severity.

	ED Severity Continuum		ED Group (>11)		ED Group (>20)	
Predictors	ß [95% CIs]	*p*	OR [95% CIs]	*p*	OR [95% CIs]	*p*
Ave. state BD	0.42 [0.23, 0.61]	<0.001	3.35 [1.78, 6.30]	<0.001	4.18 [1.65, 10.60]	0.003
Ave. state neg mood	−0.17 [−0.36, 0.02]	0.083	0.48 [0.27, 0.87]	0.016	0.38 [0.17, 0.86]	0.020
Freq. dietary restraint	0.15 [−0.007, 0.37]	0.172	1.31 [0.59, 2.92]	0.513	1.25 [0.66, 2.37]	0.502
Freq. comparisons	0.41 [−0.16, 0.98]	0.159	2.25 [0.40, 12.59]	0.355	1.58 [0.21, 11.63]	0.653
Persistence of state BD	−0.10 [−0.29, 0.09]	0.323	0.63 [0.33, 1.17]	0.145	0.83 [0.30, 2.31]	0.716
Persistence of neg mood	−0.01 [−0.18, 0.16]	0.929	1.06 [0.60, 1.87]	0.837	1.47 [0.61 3.54]	0.391
BD → comparisons	−0.06 [−0.62, 0.50]	0.827	0.75 [0.15, 3.73]	0.722	1.01 [0.14, 7.36]	0.994
BD → restraint	−0.16 [−0.52, 0.20]	0.365	0.63 [0.21, 1.86]	0.399	0.17 [0.05, 0.61]	0.006
Neg mood → restraint	0.10 [−0.31, 0.03]	0.968	0.86 [0.32, 2.32]	0.764	3.72 [1.30, 10.65]	0.015
Restraint → BD	−0.09 [−0.21, 0.03]	0.179	1.15 [0.80, 1.66]	0.457	0.74 [0.49, 1.10]	0.142
Restraint → neg mood	−0.05 [−0.19, 0.09]	0.486	0.77 [0.52, 1.14]	0.202	0.99 [0.69, 1.42]	0.952
Comparisons → BD	0.03 [−0.16, 0.22]	0.758	0.63 [0.33, 1.20]	0.160	1.61 [0.89, 2.92]	0.114
*R* ^2^	0.34		0.39		0.43	

*Note*. Ave = average, BD = body dissatisfaction, freq = frequency, neg mood = negative mood states, restraint = dietary restraint. ED = eating disorders. ED groupings based on whether an individual scores above a threshold of 11 or 20 on the EAT26. No adjustments were made for confounds.
